# Public Health Innovations on the Way to Interruption of Poliovirus Transmission in Nigeria

**DOI:** 10.1093/infdis/jiv537

**Published:** 2016-04-02

**Authors:** Rui G. Vaz, Pascal Mkanda, Peter Nsubuga, Muhammad Ado, Andrew Etsano

**Affiliations:** 1 World Health Organization, Nigeria Country Office; 2 National Primary Healthcare Development Agency, Federal Ministry of Health Nigeria, Abuja, Nigeria; 3 World Health Organization, Regional Office for Africa, Brazzaville, Congo; 4 Global Public Health Solutions, Atlanta, Georgia

**Keywords:** polio eradication initiative, public health innovations, World Health Organization, outbreak response, Nigeria

The polio eradication journey in Nigeria has been long, with a mix of good and not so good news over the years. Nigeria was very close to interrupting transmission of polio and then experienced an upsurge of transmission, owing to an interaction of several factors. At one point, Nigeria became a net exporter of poliovirus to polio-free countries, but at the end of 2013 there was clear evidence that the hard work and numerous innovations that the government and its partners had implemented were beginning to pay off: the number of cases of wild poliovirus (WPV) type 1 infection had decreased by 58%, compared with 2012; there had not been any cases of WPV type 3 infection detected since November 2012; and there had also been a reduction in the genetic diversity (clusters) of WPV1, from 8 in 2012 to 2 in 2013. In 2014, there were 6 cases of WPV1 infection, compared with 53 in 2013, and the number of genetic clusters had decreased to 1.

Poliovirus transmission in Nigeria has been driven by reservoirs in northern Nigeria, where several states are considered to be at very high risk of poliovirus transmission. Additionally, 3 states in the northeast region have suffered from insecurity, and this added another facet to the struggle to control polio; several health workers gave the ultimate sacrifice to polio eradication, when they were killed as they conducted their work.

All public health programs rely on effective surveillance and prompt response guided by the best information available. Polio eradication, like other infectious diseases surveillance and response programs, relies on prompt detection, registration, and laboratory and epidemiologic confirmation of suspected polio cases; reporting, analysis, use, and feedback of data; and preparedness and response (eg, outbreak investigations, contact tracing, and public health interventions). Like other programs, polio eradication needs managerial and support functions, which include coordination, supervision or performance evaluation, training, and resource provision for infrastructure, including communication. In Nigeria, each of these core activities and support functions were needed for the polio eradication program to produce the remarkable results that it has achieved, given the situation that it is in. New innovations also had to be tried and perfected to solve complex problems that revolved around the community accepting incessant rounds of oral polio vaccination.

This supplement presents some of the public health programmatic innovations that the Nigerian government and its polio partners, with the support of the World Health Organization, tried and implemented to interrupt the transmission of polio. The programmatic interventions that are described here include efforts to create demand for polio vaccination; strategies to detect every case of suspected WPV, including those in the environment; methods to determine the underlying population immunity; strategies to effectively plan for, implement, and track vaccination activities, using the latest technologies; and approaches to widen the polio partnership in noncompliant and security-compromised communities. These interventions were enabled by a focused effort to obtain the necessary resources, use them effectively and transparently, provide the needed public health workforce, and ensure accountability for results.

In 2014, Nigeria was one of the countries that was affected by the Ebola virus disease outbreak in West Africa. This supplement reviews how the polio partnership demonstrated remarkable flexibility by addressing and quickly overcoming this major public health challenge, using lessons and experiences obtained from polio eradication without compromising the gains that had been won for polio eradication.

At the time this supplement was prepared, Nigeria had gone 12 months with no report of WPV and had started using inactivated polio vaccine. We believe that these public health interventions and other activities will help Nigeria enter a poliovirus-free environment. We also believe that these lessons can be adapted to improve routine immunization in Nigeria. We hope you will enjoy learning about these interventions.

